# Fully automated rigid image registration versus human registration in postoperative spine stereotactic body radiation therapy: a multicenter non-inferiority study

**DOI:** 10.1093/jrr/rrab113

**Published:** 2021-12-20

**Authors:** Yutaro Koide, Hidetoshi Shimizu, Risei Miyauchi, Shouichi Haimoto, Hiroshi Tanaka, Yui Watanabe, Sou Adachi, Daiki Kato, Takahiro Aoyama, Tomoki Kitagawa, Hiroyuki Tachibana, Takeshi Kodaira

**Keywords:** spine, bone metastases, stereotactic body radiation therapy (SBRT), image registration (IR), rigid image registration (RIR), CT-myelogram, spinal cord

## Abstract

To confirm the fully automated rigid image registration (A-RIR) accuracy in postoperative spine stereotactic body radiation therapy (SBRT), we conducted a multicenter non-inferiority study compared to the human rigid image registration (H-RIR). Twenty-eight metastatic cancer patients who underwent postoperative spine SBRT are enrolled—image registration (IR) of planning computed tomography (CT) and CT-myelogram for delineating the spinal cord. The adopted A-RIR workflow is a contour-focused algorithm performing a rigid registration by maximizing normalized mutual information (NMI) restricted to the data contained within the automatically extracted contour. Three radiation oncologists (ROs) from multicenters were prompted to review two blinded registrations and choose one for clinical use. Indistinguishable cases were allowed to vote equivalent, counted A-RIR side. A-RIR is considered non-inferior to H-RIR if the lower limit of the 95% confidence interval (CI) of A-RIR preferable/equivalent is greater than 0.45. We also evaluated the NMI improvement from the baseline and the translational/rotational errors between A-RIR and H-RIR. The A-RIR preferable/equivalent was selected in 21 patients (0.75, 95% CI: 0.55–0.89), demonstrating non-inferiority to H-RIR. The A-RIR’s NMI improvement was greater than H-RIR in 24 patients: the mean value ± SD was 0.225 ± 0.115 in A-RIR and 0.196 ± 0.114 in H-RIR (P < 0.001). The absolute translational error was 0.38 ± 0.31 mm. The rotational error was −0.03 ± 0.20, 0.05 ± 0.19, −0.04 ± 0.20 degrees in axial, coronal, and sagittal planes (range: −0.66–0.52). In conclusion, A-RIR shows non-inferior to H-RIR in CT and CT-myelogram registration for postoperative spine SBRT planning.

## INTRODUCTION

Spine stereotactic body radiation therapy (SBRT) is an emerging treatment in spinal metastatic cancer patients. Numerous retrospective studies and some prospective studies show promising local control over the 3-dimensional conventional radiotherapy [1–8]. Some consensus guidelines have been published for SBRT planning, and one is about postoperative settings [9–12].

Spine SBRT planning requires accurate image registration (IR) of MRI/CT-myelogram to the planning computed tomography (CT) for spinal cord delineation. For SBRT planning, the Spine Response Assessment in Neuro-Oncology (SPINO) group recommends the IR of axial T1/T2-weighted MRI and the treatment planning CT [13]. The group also recommends CT-myelogram for spinal cord delineation in case of significant hardware artifact or MRI contraindicated situations. Thirty-eight percent of the groups’ facilities use CT-myelogram in such circumstances. In clinical practice, rigid image registration (RIR) is performed by trained staff. RIR has been for many years performed either completely manually or with manual intervention [14]. Still, it has not been tested whether automated registration can be used for spine SBRT instead of human rigid image registration (H-RIR).

We implemented a fully automated RIR algorithm of CT to CT-myelogram specifically for postoperative SBRT. In addition, we conducted a single-blinded non-inferiority study comparing automated rigid image registration (A-RIR) versus H-RIR to prove our hypothesis that A-RIR is non-inferior to H-RIR. If successful with this study, A-RIR may contribute to timesaving and reducing the burden on the medical staff.

## MATERIALS AND METHODS

### Patient selection

Our institutional review board approved this study. All participants provided written informed consent and underwent postoperative spine SBRT of 24 Gy in 2 fractions from May 2018 to April 2021. The eligible patients were all performed CT-myelogram because of the surgical hardware artifact. Surgical procedures were mainly posteriorly approached, with decompression and fixation or fixation only. The extent of pedicle screw fixation was usually two levels above and two levels below the affected vertebrae. After the surgical wound healing, patients underwent CT simulation and CT-myelogram scans as soon as possible.

### Planning CT simulation and CT-myelogram

All patients underwent planning CT scans in the immobilized supine position with an Aquilion LB CT system (Canon Medical Systems, Tochigi, Japan) under the following conditions: tube voltage = 120 kV, tube current = automatic exposure controlled, matrix size = 512 × 512 pixels, field of view (FOV) = 550 mm, slice thickness = 1 mm, the spatial resolution = 1.074 mm/pixel.

CT-myelogram was obtained through an intrathecal injection of iohexol contrast (Omnipaque 240; GE Healthcare, Princeton, New Jersey, USA), followed by CT simulation. Two hours after the injection, a CT scan is taken with the same CT system and immobilization as CT simulation. If the treating lesion is located in the cervical spine or upper thoracic spine, the patient is placed in a high pelvic position (e.g. Trendelenburg position) before imaging to allow the contrast medium to spread.

### Manual image registration

This study compared with the H-RIR results used in the actual clinical cases. Our routine IR procedure is described as follows:

1. The treating radiation oncologist (RO) makes a registration request specific to the patient defining primary/secondary image, what landmark to register to, and accuracy requirements.
2. A trained member of the radiotherapy team performs the requested registration completely manually or with assisted automated/semi-automated registration tools.
3. The treating RO evaluates and approves the registration results for clinical use.

The following conditions were requested for postoperative spine SBRT cases: The primary image is planning CT, and the secondary image is CT-myelogram. The number of treating vertebrae determines the registration landmarks. If the treating lesion is limited to one or two vertebrae, the landmarks extend to the lesioned vertebrae plus one vertebral bone and hardware of the upper/lower side (i.e. 3–4 vertebrae). If the lesion spreads to three or more vertebrae, the lesioned vertebral bones themselves are used for registration landmarks. The accuracy was required within 1 mm because the slice thicknesses of primary/secondary images were both 1 mm. The protocol determines these conditions and is also used as a reference in implementing the A-RIR.

### Fully automated rigid image registration

A fully automated RIR system was implemented with the customizing workflow of MIM Maestro® (MIM Software Inc., Cleveland, USA). We chose a contour-focused normalized mutual information (NMI) based RIR algorithm. Mutual information is commonly used in IR, defining it as ‘the amount of uncertainty about image B minus the uncertainty about B when A is known’ [15]. The NMI was proposed by Studholm *et al.* for handling the sensitivity overlap, which ranges from 0–1 and shows 1 when the two images A and B are exactly matched [16]. Mutual information is calculated on the whole image, but this contour-focused algorithm performs rigid registration by maximizing NMI restricted to the data contained within the user-specified contour. The contour used in this algorithm is automatically extracted to be in line with the landmarks (lesioned vertebrae and hardware) described in our in-house protocol. When this workflow runs, the A-RIR process can be complete within 1–2 minutes. We defined the default rigid assist alignment of MIM Maestro® as the baseline, which is NMI-based registration on the whole image. The evaluating RO can determine the optimal registration compared to baseline fused images, A-RIR images, and H-RIR images (Fig. 2). Also, we can calculate the contour-specific NMI and the actual 3D translation (translation and rotation) from the baseline.

### Blinded review and outcome

Before starting the review, we prepared masked A-RIR and H-RIR results of all patients. Each patient’s results were displayed on the horizontal split screen with automatically shuffled (Fig. 2). Three ROs (8–9 years experiences of radiation oncology) from multicenter were prompted to choose which of the two blinded registrations they preferred for clinical use. The fused images are initially displayed as an image overlay, but the evaluators can change the overlay ratio or changing it to a checkerboard display to suit their preference. If some cases look indistinguishable for evaluators, they were attempted to vote ‘equivalent.’ The selection result was the one with the most votes. If three people had different opinions (i.e. A-RIR: 1, H-RIR: 1, equivalent: 1), they were considered ‘equivalent.’ The registration selection results were recorded in all cases. The three ROs reviewed independently, and the voting results were blinded until all voting finished. Referring to the non-inferiority study design described in some papers [17, 18], we defined the ratio of the sum of ‘A-RIR’ and ‘equivalent’ votes as the primary endpoint, with the most respect to RO selection. We sought to determine whether A-RIR was not worse than H-RIR with a 5% inferiority margin. A-RIR will be considered non-inferior to H-RIR if the lower limit of the 95% confidence interval (95% CI) of the ratio of ‘A-RIR’ and ‘equivalent’ is greater than 45%.

We also calculate the local NMI and Pearson’s correlation coefficient as the quantitative evaluation. We use the contour to calculate these two indices, automatically created on the primary CT matching with the bony landmarks when the A-RIR runs.

**Table 1:** Patient characteristics

Characteristics	Data
Median age (range), years	62 (26–82)
Sex, n (%) Male Female	12 (42.9%) 16 (57.1%)
Histology, n (%) Lung cancer Gastrointestinal cancer Thyroid cancer Sarcoma Other (Breast, Kidney, Brain, Uterus)	8 (28.6%) 11 (39.3%) 2 (7.1%) 3 (10.7%) 4 (14.3%)
Median number of vertebral levels treated (range)	2 (1–5)
Location of the main treated tumor, n (%) Cervical Upper thoracic (Th1–6) Lower thoracic (Th7–12) Lumbar–Sacral	4 (14.3%) 7 (25.0%) 9 (32.1%) 8 (28.6%)
Median clinical target volume, cm^3^ (range)	91.3 (20.2–359.6)
Median time to CT-myelogram from planning-CT, days (range)	1 (−4–4)
Median spatial resolution of the axial plane (range), mm/pixel Planning-CT CT-myelogram	1.074 (0.976–1.074) 1.074 (0.272–1.074)

### Statistics

Statistical analysis and required sample size calculation were performed using R version 3.6.1 (The R Foundation for Statistical Computing, Vienna, Austria). In the absence of standard deviation and mean difference data from previous studies, a preliminary experiment was performed using 10 initial patients to calculate the sample size based on the NMI difference corresponding to the required accuracy. The corresponding NMI loss was recorded from the H-RIR results translated by 1 mm in the anterior–posterior, left–right, and craniocaudal directions or rotated by 1 degree in axial, sagittal, and coronal sections. The results showed that 95% of the data for NMI loss per 1 mm error or a 1-degree error was greater than 0.05. Therefore, the acceptable non-inferiority margin of NMI loss was set as 0.05. The standard deviation was 0.11, and the mean difference was 0.03, so the sample size was required 24 (one-sided α = 0.05, statistical power = 0.8).

A one-sided paired t-test was used for comparing the NMI improvements from the baseline registration between A-RIR and H-RIR. The threshold for statistical significance was set at *P* < 0.05. The Pearson correlation coefficient was compared in the baseline, A-RIR, and H-RIR using one-way analysis of variance (one-way ANOVA). Post-hoc Tukey’s honestly significant difference test was performed if one-way ANOVA overall has *P* < 0.05.

## RESULTS

### Patient data

Twenty-eight patients were included in this study. They all underwent the pedicle screw fixation above and below the affected vertebrae followed by the spine SBRT following the in-house protocol. Table 1 shows the patients’ characteristics. One patient was not immobilized well due to pain at the CT simulation. He underwent CT-simulation four days after CT-myelogram, and the second CT was used for planning. Four patients underwent CT with a smaller FOV than 550 mm to focus on the spinal cord.

### **Qualitative validation results (primary outcome**)

Three ROs (9-year experience: two ROs, 8-year experience: one RO) from three different facilities did the blinded review. Table 2 describes the summary of the RIR selection results. The lower limit of the 95% CI of the sum of the ‘A-RIR’ and ‘equivalent’ was 0.55, demonstrating non-inferiority (‘A-RIR’ = 17 patients, ‘equivalent’ = 4 patients, ‘H-RIR’ = 7 patients). The detailed individual selection results were described in the Supplementary Table 1.

**Table 2:** Blind review results

A-RIR chosen	Equivalent	H-RIR chosen	A-RIR chosen or equivalent (95% CI)
17	4	7	0.75 (0.55–0.89)

RIR: rigid image registration, CI: confidence interval

**Table 3:** Results of local NMI improvements and Pearson correlation coefficient

	Baseline	A-RIR	H-RIR	P
NMI improvement mean ± SD (range)	0	0.225 ± 0.115 (0.036–0.466)	0.196 ± 0.114 (0.018–0.434)	<0.001
Pearson CC, mean ± SD (range)	0.721 ± 0.278 (0.039–0.980)	0.978 ± 0.024 (0.901–0.997)	0.972 ± 0.030 (0.876–0.996)	<0.001^*1^, 0.99^*2^

NMI: normalized mutual information, Pearson CC: Pearson correlation coefficient, A-RIR: automated rigid image registration, H-RIR: human rigid image registration,

^*1^P-value result of one-way analysis of variance comparing the baseline vs A-RIR vs H-RIR

^*2^P-value result of Post hoc Tukey’s test comparing A-RIR vs H-RIR

### Quantitative accuracy of image registration

The NMI improvement of A-RIR was greater than H-RIR in 24 patients (Table 3): the mean value ± SD was 0.225 ± 0.115 in A-RIR and 0.196 ± 0.114 in H-RIR (*P* < 0.001). In addition, both A-RIR and H-RIR showed higher Pearson CC values than baseline (*P* < 0.001), but they did not show significant differences in each other (P = 0.99).

Both the translational and rotational errors were less than 1 mm or 1 degree between A-RIR and H-RIR as follows. The mean ± SD translation error was −0.14 ± 0.48, 0.16 ± 0.55, 0 ± 0.15 mm in anterior–posterior, left–right, and craniocaudal directions. The absolute translation error in the three dimensions was 0.38 ± 0.31 mm. The mean ± SD rotation error was −0.03 ± 0.20, 0.05 ± 0.19, −0.04 ± 0.20 degrees in axial, coronal and sagittal planes. The largest angular error was 0.66 degrees (range: −0.66–0.52 degrees).

## DISCUSSION

The most standard method of IR for spinal cord segmentation in spine SBRT is to fuse the planning CT and T1/T2 weighted MRI. Although MRI is recommended in most cases, CT-myelogram may be helpful in surgical hardware artifacts, or MRI contraindicated situations. Our study aimed to confirm our hypothesis that an A-RIR is not inferior to human registration in planning CT to CT-myelogram registration. Some studies [1, 9] have used simulation CT and fused CT-myelogram for treatment planning like ours, while Beeler *et al.* has planned on CT-myelogram itself [19]. A systematic review reported ±20 HU is the tolerance for soft tissue that can keep the dose change in treatment planning to <1% [20]. However, since CT-myelogram strongly enhances the surrounding area of the spinal cord, it is unclear how much the dose calculation accuracy of the spinal cord was affected.

The American Association of Physicists in Medicine commissioned Task Group 132 recommends that the IR is performed by a trained member of the radiotherapy team. Furthermore, the accuracy of registration results is recommended to be evaluated by the treating RO [14]. Our institution is following this report. They also recommend that the IR accuracy is assessed with qualitative points (e.g. split-screen and checkerboard displays, image overlay displays) and quantitative points (e.g. target registration error [TRE], mean distance to agreement). Still, most facilities tend to focus on the qualitative review [21]. With the most respect to the RO judgment for clinical use, we used a blinded comparison of two registrations by ROs as the primary endpoint. The results showed that A-RIR was non-inferior to H-RIR in the primary endpoint and showed good registration accuracy in the secondary endpoints.

**Fig. 1:**
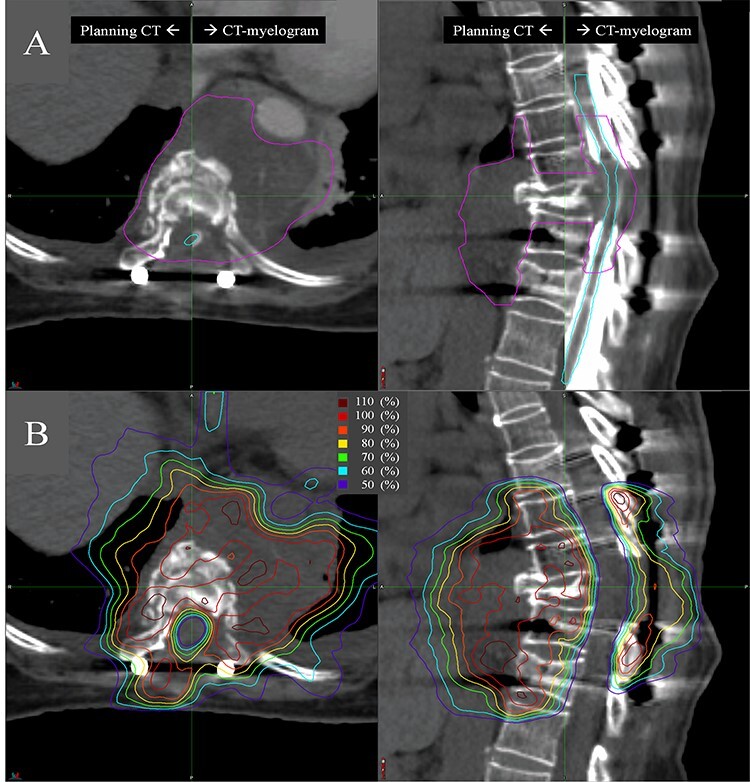
An example of treatment planning for postoperative spine SBRT. A 59-year-old woman with metastatic endometrial cancer was affected in the ninth thoracic vertebrae and surrounding epidural/prevertebral space. **A:** Fused CT images of planning CT (left) and CT-myelogram (right) in the axial and sagittal plane with delineated clinical target volume (pink) and spinal cord (light blue). **B:** Axial and sagittal plane of planning CT with dose distribution.

Although TRE is used as a quantitative measure of registration accuracy, we did not use it. The corresponding points need to be defined accurately, adequately, and appropriately for calculation, but it is challenging to set the points because the lesioned vertebral body is highly infiltrated and destructed by the tumor (Fig. 1). Therefore, we instead measured the error of the registration shift from the baseline for the A-RIR and H-RIR. In other words, we are looking at how much translation and rotation are needed to get the A-RIR to match the H-RIR perfectly. The results show that the error between the A-RIR and the H-RIR is small, less than 1 mm and less than 1 degree.

**Fig. 2:**
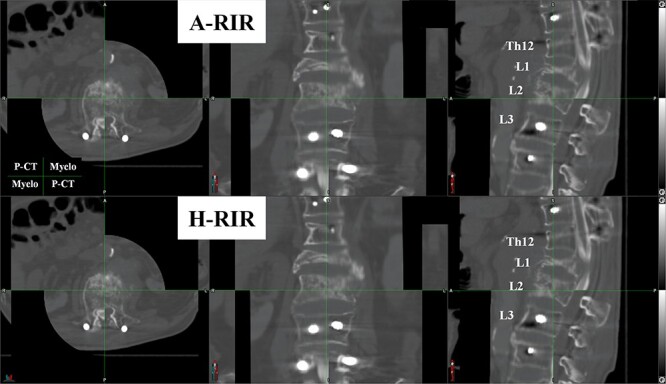
An example of the blind review comparing two registration results. Each patient’s registrations were displayed on the horizontal split screen with automatically shuffled. Three evaluators are prompted to choose the registration they would like to use clinically. The figure is shown on a checkerboard display (the planning CT: the upper left and lower right parts, the CT-myelogram: the upper right and lower left). The evaluators can review all the slices of CT images. A-RIR: automated rigid image registration, H-RIR: human rigid image registration, P-CT: planning CT, Myelo: CT-myelogram.

We also evaluated the NMI difference between A-RIR and H-RIR as a secondary endpoint. The higher NMI means the better the registration accuracy of two images. We looked at the NMI improvement from baseline within the contour containing the bony landmarks and found that the A-RIR was slightly better than H-RIR (0.23 vs 0.20, P < 0.001). The standard deviation was 0.11 in common, which means that the A-RIR can always produce similar registration results produced by trained staff, regardless of lesion location.

There are two types of IR: RIR and deformable image registration (DIR). DIR is an increasingly common method commonly used in adaptive radiotherapy with better performance than RIR [22, 23], but many facilities still only use RIR for treatment planning and irradiation. Yuen *et al.* described in an international survey in 2018 that 92–100% of facilities used RIR, and 22–77% used DIR for CT–CT registration. For CT-MR registration, 83–100% used RIR, and 0–33% used DIR. Although there is a recommendation to standardize IR for segmentation from RIR to DIR, such as head and neck cancers [22, 23], there is still no such proposal for postoperative SBRT. The possible reasons why RIR is still used for spine SBRT planning are (i) the treatment target is a rigid vertebra, (ii) the manually RIR works well in an accurate registration, (iii) DIR causes deforming the tumor or the spinal cord [14], and (iv) RIR is used in actual irradiation.

No studies are comparing A-RIR with H-RIR for spine SBRT planning. We set a strict criterion that the required accuracy is within 1 mm, and our results show that the two RIRs are clinically indistinguishable for ROs. Both planning CT and CT-myelogram are taken with 1 mm slices in our institution, so we are pursuing accuracy up to the spatial resolution limit. The required accuracy for spine SBRT is relatively high compared to the general registration requirement of 2 mm [14]. If the slice thickness exceeds 2 mm, it is difficult to evaluate whether the registration accuracy is within the recommended range. While it is recommended that a trained planner do registration, the A-RIR is accurate enough to do that task instead. Since it is fully automatic, it can be used by untrained staff.

Some significant limitations exist in this study. First, this study retrospectively compared the A-RIR to the clinically-used H-RIR. Although the accuracy of our H-RIR is used as a reference, it is not perfect and has a specific (clinically acceptable) level of error. Second, this study is based on cases with a median of two vertebrae lesions. Thus, two or more separate RIR might be needed to accomplish the clinical goal in some advanced cases (e.g. an extended lesion of more than 2–3 vertebras, multiple skipped lesions). Third, the algorism we used is still updated, which means not perfect yet. Finally, there were three cases where A-RIR showed lower NMI than H-RIR, which may be due to the algorithm.

## CONCLUSION

This study showed that A-RIR is non-inferior to the accuracy of H-RIR in CT and CT-myelogram registration for postoperative spine SBRT planning.

## Supplementary Material

Supplementary_table_rrab113

## Data Availability

Research data are stored in an institutional repository and anonymized numerical data will be shared upon request to the corresponding author. Research image data are not available at this time.
